# Young adults with depression: A registry-based longitudinal study of work-life marginalisation. The Norwegian GP-DEP study

**DOI:** 10.1177/14034948231165089

**Published:** 2023-04-17

**Authors:** Øystein Hetlevik, Tone Smith-Sivertsen, Inger Haukenes, Sabine Ruths, Valborg Baste

**Affiliations:** 1 Department of Global Public Health and Primary Care, University of Bergen, Norway; 2 Research Unit for General Practice, NORCE Norwegian Research Centre, Norway; 3 Division of Psychiatry, Haukeland University Hospital, Norway; 4 National Centre for Emergency Primary Health Care, NORCE Norwegian Research Centre, Norway

**Keywords:** Depression, comorbidity, young adult, general practice, education, employment, social security

## Abstract

**Aims:**

To explore the association between a depression diagnosis in young adulthood and risk of marginalisation at age 29 years, among those who had completed upper secondary school and those who had not completed at age 21.

**Methods:**

In a longitudinal cohort study based on nationwide registers we followed 111,558 people from age 22–29 years. Outcomes were risk of marginalisation and educational achievement at age 29. Exposure was a diagnosed depression at ages 22–26 years. Comorbid mental and somatic health conditions, gender and country of origin were covariates. Relative risks were estimated with Poisson regression models, stratified by educational level at age 21.

**Results:**

For people who had not completed upper secondary school at age 21 years, a depression diagnosis at age 22–26 increased the risk of low income (relative risk = 1.33; 95% confidence interval = 1.25–1.40), prolonged unemployment benefit (1.46; 1.38–1.55) and social security benefit (1.56; 1.41–1.74) at age 29 compared with those with no depression. Among those who had completed upper secondary school at age 21 years, depression increased the risk of low income (1.71; 1.60–1.83), prolonged unemployment benefit (2.17; 2.03–2.31), social security benefit (3.62; 2.91–4.51) and disability pension (4.43; 3.26–6.01) compared with those with no depression. Mental comorbidity had a significant impact on risk of marginalisation in both groups.

**Conclusions:**

**Depression in one’s mid-20s significantly increases the risk of marginalisation at age 29 years, and comorbid mental health conditions reinforce this association. Functional ability should be given priority in depression care in early adulthood to counteract marginalisation.**

## Background

There is a growing concern in Europe for young people who leave the education system prematurely or do not establish a place in the labour market during young adulthood [1,2]. In 2020, an average of 16% of people aged 20–24 years and 19% of those aged 25–29 years in EU countries were not in employment, education or training (NEET) [1]. The corresponding figures for Norway, which have been relatively stable in the last decade [3], were 8% and 9% among men, and 7% and 11% among women [1]. These young people are at risk of marginalisation due to poverty and the lack of skills or education to improve their situation [4,5].

Reasons why young people become part of the NEET group are no doubt complex, but mental health problems seem to be an important explanatory factor [6–8]. Depression is the most common mental health disorder in younger age and an important contributor to functional loss [9]. A large group of young people live with prolonged moderate symptoms [10,11]. Depression in adolescence is associated with adverse educational and economic outcomes in the early 20s [12] and later in adult life [13–15]. However, there is a lack of knowledge about how educational attainment in late adolescence and depression in young adulthood interact with respect to the risk of later marginalisation.

The main path into higher education or stable employment is to complete upper secondary school. Completion is thus an important societal indicator to reduce the risk of marginalisation. In Norway, about 60% complete upper secondary school in the normal length of time at age 18–19 years, while one in four people in their early 20s still have not completed [16]. Mental health problems are associated with drop-out among adolescents [17,18]. Students with substance addiction or attention deficit hyperactivity disorder (ADHD) have the highest drop-out rates, but depression and anxiety are also significantly associated with school drop-out [19–21]. Drop-out is considered an independent risk factor for later marginalisation, and measures to help adolescents complete upper secondary school are important to reduce this risk [13,17,22,23]. In the years following upper secondary school most young people start higher education or establish themselves in working life, with no need of structured societal support to help them succeed. Those who have completed upper secondary school have an advantage in this important transitional period [22,24]. However, the question arises of whether depression and other mental health problems that occur in the early 20s affect this advantage. To the best of our knowledge, no previous study has examined this issue.

Using rich data from national Norwegian registries, including information on health, education, income and social benefits, we had the opportunity to study the long-time impact of depression in young adulthood on the risk of marginalisation in a nationwide sample. The aim of this study was to explore the association between a certified depression diagnosis at age 22–26 years and the risk of low income, long-term unemployment benefits, social benefits, disability pension and further educational achievement at age 29. We stratified the study population by completion/non-completion of upper secondary school at age 21, to assess whether the possible associations were different in the two groups of educational level.

## Methods

### Setting

The Norwegian education system comprises 10 years of mandatory schooling (elementary school and lower secondary school), followed by a 3–4-year voluntary educational track at upper secondary school from age 16 to 18–19 years. However, for some pupils this educational step is prolonged and national statistics include a five-year educational track. Therefore, we chose age 21 years as cut-off in our analyses. Upper secondary schools provide either vocational training qualifying students for a practical profession, or theoretical education, which is mandatory for access to higher education (university college and university). Nine per cent of students in upper secondary schools attend private schools that are mainly public financed with a moderate fee for students.

The Norwegian Labour and Welfare Service (NAV) provides social and economic security while encouraging a transition to activity and employment, regardless of health problems. Support measures include, for example, unemployment benefits provided for shorter or longer periods, social benefits to ensure subsistence, and disability pension for longstanding serious health problems.

## Design

This study is a part of the Norwegian GP-DEP Study, a registry-based cohort study investigating pathways of depression care in general practice. The cohort is closed and includes all residents in Norway who were 12 years or older by 1 January 2008 (4,017,989 individuals). The current study sample is a cohort of people born in 1986 and 1987, chosen to examine associations between a depression diagnosis at age 22–26 years (important transitional period) and risk of marginalisation at age 29.

## Data sources

We obtained data from six national registries. The population at risk comprised all residents in Norway born in 1986 or 1987 who had lived in Norway during the whole study period from 2008 to 2016 according to the Population Registry. We obtained complete information regarding the residents’ gender, year of birth, death and emigration. From the National Educational Database, we extracted information on the highest completed educational level at ages 21 and 29 years. The Norwegian Social Insurance Database provided information on social security benefits, including social financial support, unemployment benefits and disability pension.

The Control and Reimbursement of Health Care Claims (KUHR) database stores data on all fee-for-service claims from general practitioners (GPs) and medical specialists (i.e. psychiatrist or psychologist) contracted to the public health care system. From this database we extracted information on all encounters with a recorded diagnosis of depression according to the International Classification of Primary Care 2nd version (ICPC-2) for GPs, and the International Classification of Disease 10th revision (ICD-10) for specialists. Additionally, we used ICPC-2 diagnoses codes from all GP consultations to obtain information about comorbidity.

The Norwegian Patient Registry (NPR) comprises information on all patient contacts with public specialised mental health care. We obtained information on all contacts with a recorded diagnosis of depression according to ICD-10 in psychiatric wards, as inpatients or outpatients.

The Norwegian Prescription Database (NorPD) contains information on all prescription drugs dispensed at pharmacies to individual patients treated in ambulatory care. For each prescription of an antidepressant drug, NorPD provided information on dispensing date, generic drug information (Anatomical Therapeutic Chemical (ATC) code) and any reimbursement code linked to specific diagnoses. We included all prescriptions of antidepressant drugs (ATC code N06A) reimbursed by Norwegian public insurance for the treatment of depression.

The data from the different registries were linked at the individual level by the Norwegian Institute of Public Health, using the (encrypted) unique personal ID number assigned to all residents of Norway. Data were anonymised before being handed over to the research project. The research database is stored and analysed on a secure server at the University of Bergen.

## Study population

The source population comprised all residents in Norway as of 1 January 2008 who were born in 1986 and 1987 and were still living in Norway in 2016 (*N*=115,353). Those who died (*N*=445) or emigrated (*N*= 3320) during the study period until the age of 29 years were excluded, leaving a study population of 111,588 individuals.

## Variables

### Outcome

For this study, we have defined five binary outcome measures as indicators of marginalisation and further education:

1. Low income: a taxable annual income below NOK 200,000 (€ 22,000 in 2016), which is close to the poverty line, during two consecutive years when the participants were aged 28 and 29 years.
2. Unemployment benefits from NAV received by the participants at age 27, 28 and 29, but not necessarily continuously, to include those with need for support either for a long period or repeatedly for shorter periods during these years.
3. Social financial support from NAV: receiving more than one day’s support at age 28–29 years. This benefit is restricted to people with particularly difficult personal finances, independent of health status.
4. Disability pension from NAV: receiving disability pension at age 29 years, a lasting benefit for longstanding health problems that reduce a person’s capability to participate in work-life, based on a strict medical evaluation.
5. Educational achievement at age 29 years: upper secondary school completion if not completed at age 21, and at least three years’ higher education (bachelor’s degree) for those who had completed upper secondary school at age 21.

### Exposure

Having a diagnosis of depression at age 22 to 26 years was the exposure variable. We selected everyone recorded with a diagnosis of depression based on consultations in general practice (ICPC-2 code P76 Depression in KUHR) and/or in specialised mental health care (ICD-10 codes F32 and F33 in NPR) and/or antidepressant drug dispensing for the treatment of depression (ATC N06A reimbursed by the Norwegian State in NorPD).

### Covariates

We adjusted for co-existing mental and somatic conditions based on GP-recorded diagnoses in the study population when aged 22 to 26 years. The diagnoses were derived from a list of chronic conditions established in general practice in Scotland by Barnett et al. [25], and adapted to the ICPC-2 codes used in Norwegian general practice [26]. In addition, we included hyperkinetic syndrome/ADHD as an entity. We included the most common mental health diagnoses and grouped them into the following categories: anxiety, alcohol/drug addiction, psychosis, and ADHD. We also constructed a dichotomised (yes/no) mental health comorbidity variable, set to yes if one or more of the included comorbid mental health conditions were registered. Learning disabilities and eating disorders were also recorded, but they were few and therefore not included in the analyses. The somatic conditions included were dichotomised (yes/no) for any somatic comorbidity.

We also adjusted for gender (men, women) and country of origin, divided into: (i) Norway; (ii) Europe, North America, and Oceania; and (iii) Africa, Asia, South America, and others.

### Analyses

Descriptive statistics were used to outline the characteristics of the whole study population and stratified on completion of upper secondary school at age 21 (yes/no). Also, characteristics were provided for those with a depression diagnosis registered at age 22–26 years.

Poisson regression with robust variance estimates was used to estimate relative risk (RR) and 95% confidence interval (CI) for the association between having a depression diagnosis at age 22–26 years and being at risk of marginalisation at age 29, according to the five defined outcomes (for some measures, information at ages 27 and 28 was also included). Crude estimates are presented for the impact of depression. We further present multivariable models, first adjusting for somatic comorbidity, gender and country of origin (model 1); and thereafter additionally also adjusting for mental health comorbidities (model 2). We repeated the multivariable models using the dichotomous variable for mental health comorbid conditions as adjusting variable. Based on these models, we estimated predicted probabilities for the outcomes for depression without comorbid mental health conditions, and depression combined with any other mental health condition. All analyses were stratified by completed or non-completed upper secondary school at age 21 years and all analyses were performed by STATA version 17 (StataCorp. 2021. Stata Statistical Software: Release 17. College Station, TX: StataCorp LLC).

### Ethics approval

The study protocol has been approved by the Regional Committee for Medical and Health Research Ethics (REC) West (2017/934) and by the Norwegian Data Protection Authority (17/01372).

## Results

In the study population (*N*= 111,588), 51.2% were men, 86.7% of all participants were born in Norway and 32,947 (29.5%) had not completed upper secondary school at age 21 years (57.9% men) (Table I). A depression diagnosis was recorded for 14,865 (13.3%) individuals in the total study population at age 22–26, 21.7% of those who had not completed upper secondary school at age 21 and 9.8% of those who had completed.

**Table I.** Characteristics of the Norwegian population born in 1986 or 1987 and living in Norway at age 22 to 29 years, total and stratified by completed upper secondary school at age 21, and a subpopulation with a depression diagnosis at age 22 to 26 years.

			Completed upper secondary school at age 21					
	Whole population		No^a^		Yes		Depression	
	*N*	%	*n*	%	*n*	%	*n*	%
Total number	111,588	–	32,947		78,641		14,865	
**Gender**								
Male	57,163	51.2	19,080	57.9	38,083	48.4	5539	37.3
Female	54,425	48.8	13,867	42.1	40,558	52.6	9326	62.7
**Country of origin**								
Norway	96,716	86.7	26,495	80.4	70,221	89.3	12,899	86.8
Europe/North America, Oceania	7943	7.1	2722	8.3	5221	6.6	1022	6.9
Asia, Africa, South America, others	6929	6.2	3730	11.3	3199	4.1	944	6.3
**Registered morbidity at age 22–26**								
Depression^b^	14,865	13.3	7164	21.7	7701	9.8	–	100
Other mental health conditions^c^								
Anxiety	5562	5.0	2816	8.6	2746	3.5	2886	19.4
Addiction related diagnoses	2153	1.9	1697	5.2	456	0.6	1034	7.0
Psychosis	1393	1.3	857	2.6	536	0.7	953	6.4
ADHD	2215	2.0	1586	4.8	629	0.8	968	6.5
Any of the other mental conditions	9691	8.7	5682	17.3	4009	5.1	4772	32.1
Somatic health conditions^c^	35,121	31.5	12,367	37.5	22,754	28.9	6535	44.0

a Including those with missing data on education (*n*= 1576).

b Based on depression diagnosis in general practice and/or specialist care and/or dispensed antidepressant drug.

c Based on general practitioner consultations only.

ADHD: attention deficit hyperactivity disorder

Other mental illnesses recorded by GPs were more common among those who had not completed upper secondary school at age 21 years compared with those who had. Among those with a depression diagnosis, anxiety was the most frequent comorbid mental health condition (19.4%), while 6–7% had an addiction-related diagnosis, psychosis or ADHD.

Markers of marginalisation were more frequent among those who had not completed upper secondary school compared with those who had graduated (Table II).

**Table II.** Distribution of markers of marginalisation and education at age 29 years^a^ stratified by completed upper secondary school at age 21 or not, total and by a depression diagnosis at age 22 to 26 years or not.

	Total		Depression			
			Yes		No	
Markers of marginalisation	*N*	%	*n*	%^c^	*n*	%^c^
**Not completed upper secondary school at age 21** ^b^	32,947		7164		25,783	
Low income (<NOK200,000) at age 28 and 29	6801	20.6	2065	28.8	4736	18.4
Any unemployment benefit at age 27, 28 and 29	6710	20.4	2181	30.4	4529	17.6
Social security benefit at age 28 or 29	1811	5.5	630	8.8	1181	5.6
Disability pension at/before age 29	1274	3.9	400	5.6	874	4.6
Completed upper secondary school at age 29^c^	8904	27.0	1586	22.1	7318	28.4
Bachelor’s degree at age 29	2707	8.2	411	5.7	2296	8.9
**Completed upper secondary school at age 21**	78,641		7701		70,940	
Low income (<NOK200,000) at age 28 and 29	7698	9.8	1315	17.1	6383	9.0
Any unemployment benefit at age 27, 28 and 29	5673	7.2	1422	18.5	4251	6.0
Social security benefit at age 28 or 29	453	0.6	162	2.1	291	0.4
Disability pension at/before age 29	231	0.3	106	1.4	125	0.2
Bachelor’s degree at age 29	48,738	62.0	7164	52.9	44,663	63.0

a All Norwegian residents born in 1986 or 1987 and living in Norway at age 22 to 29 years (*N*= 111,588).

b Including those with missing data on education (*n* = 1576).

c Percentage among those with depression or no depression respectively.

### Depression as a risk factor for marginalisation

A depression diagnosis at age 22 to 26 years increased the RR of all marginalisation measures and decreased the RR of achieved education (Table III). The estimates attenuated somewhat in the adjusted analyses, especially when comorbid mental health conditions were included in the models.

**Table III.** The likelihood of marginalisation^a^ and further education^b^ at age 29 years after depression at age 22–26 years, stratified by upper secondary school completion at age 21. Based on Norwegian population born in 1986 or 1987.

	Markers of marginalisation									
	Low income		Unemployment benefit		Social security benefit		Disability pension		Further education^b^	
	RR^c^	95% CI	RR^c^	95% CI	RR^c^	95% CI	RR^c^	95% CI	RR^c^	95% CI
**Upper secondary school completion at age 21**										
**Not completed (*n*= 32,947)**										
Crude model: depression	1.57*	1.49, 1.65	1.73*	1.65, 1.82	1.92*	1.74, 2.12	1.65*	1.46, 1.85	0.78*	0.74, 0.82
Adjusted model 1: depression^d^	1.52*	1.44, 1.60	1.63*	1.54, 1.71	2.04*	1.85, 2.25	1.62*	1.44, 1.83	0.76*	0.72, 0.80
Adjusted model 2: depression^e^	1.33*	1.25, 1.40	1.46*	1.38, 1.55	1.56*	1.41, 1.74	1.12	0.98, 1.28	0.86*	0.82, 0.91
**Completed (*n*= 78,641)**										
Crude model: depression	1.90*	1.79, 2.01	3.08*	2.90, 3.27	5.13*	4.23, 6.22	7.81*	6.03, 10.1	0.84*	0.81, 0.87
Adjusted model 1: depression^d^	1.89*	1.78, 2.01	2.51*	2.36, 2.67	5.14*	4.22, 6.27	7.35*	5.63, 9.59	0.80*	0.78, 0.83
Adjusted model 2: depression^e^	1.71*	1.60, 1.83	2.17*	2.03, 2.31	3.62*	2.91, 4.51	4.43*	3.26, 6.01	0.84*	0.81, 0.87

a (i) Income below NOK 200,000 at age 28 and 29 years; (ii) unemployment benefit at age 27, 28 and 29; (iii) social security benefit at age 28 or 29; (iv) disability pension at age 29.

b Completed further education at age 29 years, that is, upper secondary school for ‘drop-outs’ and bachelor’s degree for the others.

c Relative risks (RR) for marginalisation/further education after depression (reference: persons with no diagnosis of depression at age 22 to 26 years).

d Adjusted model 1: adjusted for gender, country of origin and somatic comorbidity.

e Adjusted model 2: as model 1 but also adjusted for mental comorbidity.

* *p* <0.001

CI: confidence interval

In the group who had not completed upper secondary school at age 21 years there was an increased adjusted RR for low income (RR=1.33; 95% CI 1.25–1.40), unemployment benefit (RR=1.46; 1.38–1.55), social security benefit at age 29 (RR=1.56; 1.41–1.54), while the RR for completing further education was lower among those with a depression diagnosis aged 22–26 compared with those without a depression diagnosis.

The same pattern was seen among those who had completed upper secondary school at age 21 years, and the risk of being in receipt of a disability pension was higher among people with a depression diagnosis compared with those without depression. The relative risk of depression-related marginalisation was higher among those who had completed upper secondary school at age 21 compared with those who had not graduated, especially regarding social security benefit and disability pension.

In Table IV, predicted probability of marginalisation or further education is shown in the percentages of people with no depression diagnosis, depression without mental health comorbidities, and depression with mental health comorbidities, respectively; given all other variables held at average in the regression model used to estimate the predicted probabilities. The probability of marginalisation was significantly greater when depression was accompanied by other mental health diagnoses.

**Table IV.** Predicted probability^a^ for markers of marginalisation^b^ and further education^c^ at age 29 years, comparing young adults without depression with those with depression with and without mental comorbidity^d^ at age 22 to 26 years, stratified by upper secondary school completion at age 21.

	Low income		Unemployment benefit		Social security benefit		Disability pension		Further education^c^	
	%	95% CI	%	95% CI	%	95% CI	%	95% CI	%	95% CI
**Upper secondary school completion at age 21**										
**Not completed (*n*= 32,947)**										
No depression, no comorbidity	17.0	16.6–17.6	16.4	15.8–16.9	3.8	3.6–4.1	2.8	2.6–3.0	30.0	29.3–30.7
Depression, no comorbidity	24.6	23.1–26.0	27.8	26.5–29.1	7.7	7.0–8.4	4.1	3.5–4.7	25.0	23.5–26.5
Depression with comorbidity	33.7	31.5–35.8	32.3	30.2–34.3	13.3	11.9–14.7	7.7	6.7–8.7	16.6	15.1–18.1
**Completed (*n*= 78,641)**										
No depression, no comorbidity	8.8	8.6–9.0	5.9	5.7–6.1	0.4	0.3–0.4	0.1	0.1–0.2	63.6	63.0–64.2
Depression, no comorbidity	15.9	13.6–17.0	13.8	11.8–14.8	1.6	1.2–1.9	0.8	0.6–1.1	52.9	51.0–54.7
Depression with comorbidity	20.5	18.5–22.6	20.4	18.5–22.2	3.8	3.0–4.8	2.8	2.0–3.5	44.4	41.5–47.3

a Predicted probability for the outcomes, given all other variables in the model are held at mean, based on the fully adjusted models presented in Table III.

b (i) Income below NOK200,000 at age 28 and 29 years; (ii) unemployment benefit at age 27, 28 and 29; (iii) social security benefit at age 28 or 29; (iv) disability pension at age 29.

c Completed further education at age 29, that is, upper secondary school for ‘drop-outs’ and bachelor’s degree for the others.

d Mental comorbidity includes anxiety, addiction problems, psychosis or attention deficit hyperactivity disorder as presented in Table I, adjusted for gender, country of origin and somatic morbidity.

## Discussion

This longitudinal study following a nationwide cohort of young adults over eight years adds new knowledge of the associations between having a depression diagnosis in young adulthood (age 22 to 26 years) and risk of marginalisation at the end of the 20s. Among those who had not completed upper secondary school at age 21, a depression diagnosis increased the risk of low income, prolonged unemployment benefit, and social security benefit at age 29. Among those who had completed upper secondary school at age 21, the proportion of marginalised was lower. However, the gap in relative risk of marginalisation between those with a depression diagnosis versus those with no depression was larger. The predicted probabilities for marginalisation were higher when depression was accompanied by other mental health conditions, as compared with depression alone.

In a systematic review and meta-analyses, Clayborne et al. showed that adolescent depression was associated with failure to complete school and later unemployment [14], supported by recent Norwegian and Australian studies [18,27]. Therefore, a higher prevalence of diagnosed depression in the drop-out group and a higher absolute risk of later marginalisation compared with those who completed upper secondary school at age 21 years, found in the present study, was not surprising. To our knowledge, the study by Gibb et al. (2010) is the only previous study on the effect of mental health problems in young adulthood on further educational or work-life achievements using similar age spans as our study. In a longitudinal cohort study among nearly 1000 young adults they showed significant impact of mental health problems in the early 20s on workforce participation and income as outcomes at age 30 [15]. In contrast to our study, they found no significant impact of educational achievements.

We found, somewhat surprisingly, that having a diagnosis of depression at age 22–26 years implied a higher relative risk of later marginalisation in the group who *had* completed upper secondary school at age 21 compared with those who *had not* completed. Due to the often long-lasting and recurrent course of this disease [10,11], these young adults may have received support to complete school, but not necessarily to improve their mental health [22], and without further support and follow-up they were less able to cope in working life. Alternatively, they may suffer from a recurrent depression episode with adverse consequences for functioning, due to higher external expectations of coping with life after completing school. This finding calls for further research to explore the impact of depression at different ages across educational status.

It is well documented that NEET status in young adulthood increases the future risk of marginalisation. A Swedish study found that poor labour market attachment in the 20s increased the risk for marginalisation in later adulthood and suggested long-term follow-up to support establishment in work-life [4]. British studies showed similar results but found a benefit of completing educational steps also after upper secondary school [5,28], supporting a strategy for helping young adults also to complete higher education. Mental health problems are underlying factors for NEET status, and are potentially modifiable through societal measures and professional help from health services; however, there are still questions concerning the best suited treatment for depression in young adults [29]. Even if the symptoms of depression are reduced, there seems to be a prolonged period with reduced functioning [30]. According to our findings, more effort should be invested on improving the functional level of depressed young adults to enable them to continue education or establish in work-life. In a scoping review, Gmitroski et al. found that better integrated health and social services and sustained support, also after entering work-life, could help to establish stable employment [31]. Personal follow-up linked to employment has been proven to be useful for vulnerable groups in a Norwegian setting [32].

## Strengths and limitations

This study is based on national registries ensuring representativeness and eliminating recall bias. Data from six health, welfare and population registries linked at the individual level gave us a broad foundation for the estimates of depression and depression care. Another strength is the longitudinal design, following the study population over eight years.

We defined measures for risk of marginalisation using objective and complete information from administrative databases. These markers of marginalisation might, however, be somewhat imprecise and could include people who are not at risk. For instance, low income or low education levels might reflect personal choices rather than reduced possibilities in some subjects. However, this is less of a problem concerning the markers based on social benefits because these are means-tested.

We used depression-related contacts recorded by both GPs and mental health care specialists, as well as information on antidepressant drug prescriptions, to define diagnoses of depression. A weakness in the data on comorbidity is that information on comorbid mental illness was incomplete in the NPR, and thus comorbidity was defined by GP-recorded diagnoses only. Still, we consider that diagnoses from GPs over a five-year period give comprehensive information on comorbid mental diseases, sufficient to draw conclusions about their impact with respect to marginalisation.

We are aware of other factors that can have a major impact on risk of marginalisation, such as family support and parents’ education and economic situation. However, this information was not available in this study.

The results of this study could be transferable to countries with comparable education, health and welfare systems, such as the Nordic countries, the UK and the Netherlands. They are less comparable for countries with different organisational systems and financing.

## Conclusion

Depression among young adults (ages 22–26 years) was significantly associated with an increased risk of marginalisation at age 29, with a lower absolute risk but a higher relative risk in the group that had completed upper secondary school at age 21. The risk increased further when depression was accompanied by other mental health conditions. To counteract marginalisation at an important transition age, research on measures to help depressed young adults improve their level of functioning should be given high priority.
